# Functional Analytic Multisensory Environmental Therapy for People with Dementia

**DOI:** 10.1155/2012/294801

**Published:** 2012-01-17

**Authors:** Jason A. Staal

**Affiliations:** Division of Biological Psychiatry, Department of Psychiatry, Beth Israel Medical Center, Albert Einstein College of Medicine, First Avenue at 16th Street, 6th Floor, Silver Building, Room 34, 6 Karpas Pavilion, NY 10003, USA

## Abstract

This paper introduces Functional Analytic Multisensory Environmental Therapy (FAMSET) for use with elders with dementia while using a multisensory environment/snoezelen room. The model introduces behavioral theory and practice to the multisensory environment treatment, addressing assessment, and, within session techniques, integrating behavioral interventions with emotion-oriented care. A modular approach is emphasized to delineate different treatment phases for multisensory environment therapy. The aim of the treatment is to provide a safe and effective framework for reducing the behavioral disturbance of the disease process, increasing elder well-being, and to promote transfer of positive effects to other environments outside of the multisensory treatment room.

## 1. Introduction

The behavioral and psychological symptoms of dementia (BPSD) comprised of aggression, apathy, psychosis, depression, and increases in motor activity [1] causes much suffering for afflicted elders and their families and challenge health care systems which provide treatment. Traditional psychiatric care for BPSD has begun to incorporate aspects of three nonpharmacologic models of care: environmental vulnerability/reduced stress threshold, unmet needs, and behavioral/learning [2]. The focus of this paper can be categorized as integrating two nonpharmacologic models, environmental and behavioral/learning theory, for use with an elder with dementia in a MultiSensory Environment (MSE). An MSE is a designated space or room designed to stimulate the senses, visual, auditory, tactile, and olfactory via equipment which produces contrived reward. Two theoretical conceptualizations exist to date to explain the underlying mechanism of action of the MSE, one behavioral and the other neurological. The behavioral position, which is built upon in this paper, is that the MSE provides the elder with noncontingent sensory reinforcement which evokes states of reward and the relaxation response [3]. The hypothesized mechanism of action contrived rewards produce positive emotion which in turn increases attending to the environment, thus reducing apathy and increasing redirection of behavior and evoking the relaxation response, which is an incompatible physiological response to agitation, especially when combined with psychotropic medications [4]. The rival theory conceptualizes the MSE as an activity-based intervention that changes negative sensory experiences with sensory calming activities, thus returning a person to a more balanced level of sensory functioning, sensoristasis [5, 6]. Recent well-designed MSE studies conducted by three independent research teams in skilled nursing homes, hospitals, and acute care inpatient psychiatry hospitalization reveal reductions in apathy, agitation, aggression, depression improvement in activities of daily living, in functional performance, and improvements in well-being and interpersonal relatedness in elders with dementia [4, 5, 7].

 The research on Multisensory Environments (MSEs) has been focusing on outcomes for people with dementia which is central in defining MSEs as an empirically valid non pharmacological evidence-based treatment for reduction of the behavioral disturbances and improved quality of life associated with moderate to severe stages of the illness [4, 5, 8, 9].

To date, very little has been written on assessment, case formulation, and treatment implementation within a MSE [10]. As a result, MSEs have a black box connotation, whereby nobody seems to know what occurs in a MSE as per therapeutic process, only that it works. The rationale for this paper is to put forth a therapeutic frame work, functional analytic multisensory environmental therapy (FAMSET) that is based on behavioral theory and practice, addressing assessment, in-session treatment, the techniques that are used in session, including the interpersonal interaction between clinician and elder and integrates existing humanistic approaches to dementia care [11, 12]. This paper is the first attempt to divide the use of an MSE into two distinct types' passive and active. Passive use is exposure to an MSE whereby responses of a person are not mediated by a clinician, occupational therapist or nurse, versus an active treatment model (FAMSET) which utilizes the reward and relaxation response as a precursor for utilizing the interpersonal interaction between the elder with dementia and the clinician.

**FAMSET: Overview of Treatment**

Assessment module:
 (i) functional analysis,
 (ii) case conceptualization;
MSE assessment:
 (i) sensory profile,
 (ii) PAL,
 (iii) In-session sensory preference assessment,
 (iv) assess CRBs and CRB2s;
Post-MSE assessment:
 (i) graded introduction to MSE over time;
In-session treatment:
 (i) following and directing module,
 (ii) exploration module,
 (iii) use of confirmation interaction technique,
 (iv) random interaction module,
 (v) color, form, aesthetic response module,
 (vi) calm and secure module,
 (vii) directed interaction module,
  (a) directed technique: link build,
  (b) directed technique: build link,
 (viii) interpersonal interactive module.

## 2. Assessment

Assessment commences with contextual observation of clinically relevant behaviors (CRBs) [13] which are identified based on antecedents and the consequences of the behavior for the person with dementia [14]. The case conceptualization identifies variables that may be amendable to intervention for MSE work or a non-MSE intervention. For example, a person with dementia, his/her behavioral disturbance may present as a distorted topography which reveals an unmet need and could lead to a non-MSE intervention [15]. This clinical choice point may include initiation of the use of an MSE to assess if noncontingent sensory reinforcement can inhibit or modify the CRB in the person with dementia [10]. It is theorized that the active agent in producing change in the MSE is noncontingent sensory reinforcement which evokes states of reward and the relaxation response. In turn, it is the relaxation response which reduces physiological reactions of stress and the noncontingent sensory reward that calms the mind via distraction while increasing attending behavior to the external environment [16]. MSE preference assessment [10] can include the use of the PAL [17] to assess the functional stage of disease process in the person with dementia, (planned, exploratory, sensory, reflex) or the Global Deterioration Scale [18] and a sensory profile assessment [5]. The combination of these assessments will help structure the MSE sessions to maximize current functioning and reduce frustration in the elder and reduce the probability of a sensory mismatch which can lead to a negative outcome (placing a low sensory seeking person in a high intensity sensory situation).

FAMSET is a modular framework, breaking down the therapy into separate treatment categories. The concept behind the modular approach is to provide a pacing to the treatment that is sequential, honoring the different types of experiences that occur in a MSE from the perspective of the person receiving treatment and for the clinician to observe behavior change in the person with dementia, thus allowing the clinician to note CRBs and to shape new behavioral responses [19]. Despite the dire cognitive consequences of dementia, people with dementia can still learn [20] in relation to classical and operant conditioning [21], thus shaping and desensitization are valid approaches to behavior change [22].

After contextual observation, understanding the functional level and sensory profile of the elder, an MSE stimulus preference assessment [10] is recommended to match the stimuli of the multisensory treatment package (visual, auditory, olfactory) to create a rewarding individualized experience for the person with dementia. Multisensory may be a misleading term in relation to the amount of visual or other stimuli, for example, some people respond positively to one piece of equipment such as a bubble tube matched with a preferred type of music, while others may like to all available pieces of visual equipment combined with preferred music and scent.

During stimulus preference assessment, the CRBs of the elder, determined beforehand by the use of contextual observation of the person, are now observed by the clinician to assess changes in the CRBs displayed the elder, which may modify based on the introduction of a single MSE modality (music) or different pairings of the multisensory treatment package (music, aroma, and colored light spray). Positive changes in the elder's behavior are termed improved clinically relevant behaviors (CRB2s) [23]. The constructs of CRBs and CRB2s have a positive priming effect in the clinician, whereby the clinician is more likely to spot the CRB and socially reward behavior change, CRB2s [24].

(1) $$\begin{array}{cc} \text{Functional Analysis-}\left(\text{choice point}\right)\to \text{MSE}\;\text{Assessment}\;(\text{Sensory}\;\text{Profile},\;\text{PAL}\to \text{In-Session}\;\text{MSE} & \\ ↓\;\;\;\;\;\;\;\;\text{Assessment}\;\text{Asses}\;\text{CRBs}\;\text{and}\;\text{CRB2s})\;\;\;\;\; & \\ \text{Non-MSE}\;\text{intervention}.\;\;\;\;\;\;\;\;\;\;\;\;\;\;\;\;\;\;\;\;\; & \end{array}$$

Graded introduction to the MSE is accomplished poststimulus preference assessment. The elder is introduced to the MSE using fixed intervals of time, comprised of five minute intervals, stopping each session before the person appears satiated by the experience. The terminal time of the MSE session is between 20 and 30 minutes based upon the needs of the person with dementia. During stimulus preference assessment, CRBs and CRB2s are the primary focus of observation. Assessment is ongoing as new CRBs may develop in the elder. The nature of a progressive illness is that it may cause dips in functioning, and people with dementia are susceptible to other medical issues such as urinary tract infections which may first be observed as behavioral change but require medical intervention. Unintended outcomes, although less likely to occur based on the stimulus preference assessment, can still occur in the MSE. Continued assessment may reveal that a person with dementia does better at a certain time of day or after other activities of daily living, for example after breakfast. During the graded introduction phase of treatment the clinician begins to utilize interpersonal techniques to increase connectedness [11] between the person with dementia and the clinician and the occurrence of CRB2s. The clinician, based on a functional understanding of the person with dementia, addresses the interpersonal constructs of validation, affirmation and nurturance, combining humanistic and behavioral approaches to strengthen CRB2s [25].

Post-MSE assessment → graded introduction to MSE, using 5-minute intervals of time over several short MSE sessions, the person's dose response is established, usually between 20 and 30 minutes, is optimal time in the MSE, which can be administered like a dose ranging from 1 to three sessions or doses per week. Assessment and observation of contingent sensory reward on elders CRBs and the development of CRB2's in relation to the environment and the clinician are strengthened through social reinforcement by the clinician.

## 3. In-Session Treatment

### 3.1. Following and Directing Module

Following is similar to the enabling approach advocated by early developers of snoezelen for children with developmental disabilities such as autism [26], whereby the person with dementia leads the MSE session and the clinician follows the behavior of the person. However, due to the level of avolition associated with later stages of a neurological degenerative illness, the enabling approach may not be recommended for people with dementia [27]. Directing is a technique to provide a verbal behavioral structure to evoke doing, not commanding, a person to do something. People with dementia may need directing by the clinician to evoke behavioral activation. Orienting is a subskill of directing, whereby the clinician assists the person with dementia to adjust to the MSE and provides active guidance. Directing is also an assessment process for the clinician to note the effects of social reinforcement on the person with dementia and the functional relationship between the person's CRB and social reinforcement (validation, affirmation, and nurturance). In practice, both directing and following can be used as complementary approaches. For example, a person with dementia may be directed to look at a bubble tube in relation to movement; the person may speak about the color of the bubbles to which the clinician applies social reinforcement by validating the person with dementias response to color. Improvements in the person with dementia's behavior CRB2s are to be observed and socially rewarded regardless of who is initiating, clinician or person, with dementia (following or directing).

Directing → the clinician verbally guides the person with dementia, orienting them to the MSE, while observing CRBs and new CRB2s from the elder.
Following → the clinician follows the lead of the elder with minimal prompting by the clinician, noting the relationship between sensory exposure and CRBs and CRB2s.
Reciprocal interactions between clinician and elder may occur, vacillating between Directing *↔* Following, the clinician observes and socially rewards changes between CRBs and CRB2s in the elder as they occur.

### 3.2. Exploration Module

Exploration is a module which occurs early in MSE treatment. The person with dementia and the clinician may function along a reciprocal continuum between following and directing in this early module. The MSE is a novel environment which uses contrived rewards (light equipment) and naturally occurring reward (the interaction between the clinician and the person with dementia). Exploration is a natural extension of the human need for autonomy and change within the MSE, exploration is a means by which one investigates the world around him or her.

Reciprocal directing and Following techniques are used by the clinician as the person with dementia explores the MSE. The clinician observes changes in CRBs and the development of CRB2s as the person explores the MSE and interacts with the clinician as he or she uses humanistic approaches (validation, affirmation, and nurturance) during interactions with the elder.

### 3.3. Confirmation Interaction Technique

In the exploration phase the clinician may introduce a technique called confirmation interaction. This is a communication between the clinician and the person with dementia that provides social reinforcement to his or her verbal behavior and or nonverbal behavior. For example, if the person with dementia in his or her exploration of the MSE says “oh that is beautiful,” a confirmation response from the clinician to them would be “yes, it does look beautiful.” This Confirmation interaction may evoke more verbal behavior in the person with dementia. During this module, the clinician is assessing CRBs and CRB2s for change. A confirmation response from the clinician can enhance the effect of noncontingent sensory reinforcement across all modules (assessment and treatment) by providing one's impression of which stimuli in the MSE evoke CRB2s in the person with dementia, hence, strengthening the elder's response. This is a central focus of the confirmation interaction.

Exploration module: the confirmation interaction technique to assess the benefits of social reinforcement by the clinician to the person with dementia to strengthen the effects of noncontingent reinforcement evoked by the equipment in the MSE, to assess the benefits of the MSE and the social reward provided by the clinician to evoke and strengthen CRB2s.

### 3.4. Random Interaction Module

During this module the person with dementia is actively looking at the preferred MSE stimuli of his/her choice. During this module the clinician's confirmation interactions strengthen the association between stimuli within the MSE, including the clinician, that evoke CRB2s. For instance, a clinician may say, “I notice that as you explore this light spray, you seem more relaxed.” To strengthen interpersonal connectedness a clinician can affirm the person with dementia's response by saying “when you listen to the music and look at the galaxy panel you seem more comfortable with me next to you.”

In the random interaction module, the person with dementia is actively looking or touching in a random manner rewarding stimuli of the MSE. The confirmation interaction technique is used to strengthen noncontingent reward and CRB2s including interpersonal distance (approach versus avoidance) between the person with dementia and the clinician if that is a CRB2 of interest.

### 3.5. Color, Form, Aesthetic Response Module

Color, form aesthetic response module can occur during exploration or random interaction phases of treatment [28]. During this module the person with dementia is attending to stimuli based on the color, form, or the aesthetic representation of MSE stimuli. The person may verbalize statements such as “oh that looks so beautiful” or “this is like a ballroom.” As in prior modules, the clinician validates and affirms the person's responses. For instance in reply to a comment of beauty, a clinician may strengthen the person with dementia's verbal behavior by saying “yes it looks very beautiful indeed.”

Validation and affirmation of the perceptions of the person with dementia are strengthened using the technique of confirmation interaction. This approach may increase verbal behavior of the person with dementia.

### 3.6. Calm and Secure Module

The calm and secure module occurs once the person with dementia has habituated to the MSE. Based on operant and classical conditioning, the person has learned what to expect in the MSE. The person with dementia has moved through the modules of exploration and random interaction. Observable behavior in the person with dementia may change toward the clinician outside of the MSE at this stage of treatment. The clinician may be seen, behaviorally, as a discriminative stimulus, whereby the presence of the clinician is perceived as signal that reward is forthcoming (going to the MSE). Confirmation interactions are aimed at strengthening this connection and looking for CRB2s which may occur in the presence of the clinician outside of the MSE. For example, a CRB in a person with dementia may be agitation to which approaching him or her may be challenging. If the person with dementia is less agitated (CRB2) upon seeing the clinician, he/she is encouraged to strengthen this CRB2. For example, the clinician might say “Good to see you today, you seem to be feeling more comfortable in seeing me, shall we go to the MSE.” If the CRB is avolition and the clinician observes more active looking at the clinician by the person with dementia (CRB2) the clinician may respond “hello (Mr. or Mrs.) you seem to be making good contact with me, let us go to the MSE”. In the MSE the person with dementia is observably more comfortable, instead of exploration or random interactions; the person begins to display evidence of the relaxation response. Confirmation interactions strengthen the relaxation response. For instance, interpreting observable contingent relationships, “you seem to feel relaxed (CRB2) and feel secure when you hear (name of song) and when you look at the colored light spray in my presence.”

The clinician uses the confirmation interaction approach to strengthen or shape observable CRB2s, in particular, the relaxation response and positive interpersonal changes in the elder with dementia in relation to the clinician.

### 3.7. Directed Interaction Module

Directed interaction is a module used to increase attention to rewarding stimuli in the MSE. The clinician in a positive manner directs the person with dementia's attention to aspects of the MSE that the person is already focusing on. Confirmation interactions strengthen what an elder is paying attention to. For instance, “you look like you are enjoying watching the movement of color across the wall.”

 A second phase of this module is directing the person with dementia's attention to a part of the environment that he/she is not paying attention to. The aim of this confirmation interaction is to increase attention to the environment by shifting awareness from stimuli the person is focusing on to aspects of the environment that he/she is not. A new technique is to provide the person with dementia with a link. A link is a verbal communication from the clinician to the person with dementia that validates the person's preferences and connects the person's attention to similar or analogous stimuli in another location of the MSE. For instance, a clinician may respond “you seem to like the movement of yellow bubbles, have you noticed on the other side of the room (pointing) that there is a similar pattern of yellow and movement?”.

The directed interaction module is comprised of two phases; the first is to strengthen the person with dementia's attention to the environment using confirmation interactions. The second is slowly shifting the person's attention via direction to aspects of the environment that are not attended to in order to increase contact with the external environment. The new technique, termed a link is used by the clinician to help the person with dementia bridge the shift of attention from one aspect of the environment he/she is attending toward other stimuli in the environment by linking the rewarding stimulus aspects of the environment together. The clinician is observing CRBs and CRB2s during this process for strengthening and shaping of new behavior.

### 3.8. Directed Technique: Link Build

A clinician directed link build is a technique designed to socially strengthen responding to stimuli by expanding on a verbal comment or nonverbal behavior from a person with dementia, and linking it to a stimulus that he/she has not been attending to. For example, “you seem to enjoy the way the colored light spray looks when it is green, do you enjoy when it turns silver and sparkles, wait here it comes now.” Link builds can follow color movement and texture. For example, “this (tactile stimuli) seems to feel very smooth which you enjoy can you find any others (tactile stimuli) that feel as soft as this.” Link builds may need to be deployed slowly; the rationale is to enhance attention and concentration to the environment not to create unrealistic demands on the person.

The link build technique is designed to shape verbal or nonverbal behavior in order to increase awareness of the external environment. The clinician socially shapes behavior by strengthening CRB2s.

### 3.9. Directed Technique: Build Link

Build links can be used to promote transfer of learning by building and linking stimuli in the MSE to stimuli outside of the MSE. For example, upon exiting the MSE the clinician uses a build link by directing the person with dementia's attention to a painting on the wall of the facility. “Look at this painting, how beautiful, I see the color yellow like we just saw in the MSE in the fields near the tree do you notice this as well?” Build links can be used to strengthen CRB2s. For example, after an MSE session, the person with dementia appears calmer (CRB2) and allows the clinician to walk closer to the person or allows a nurse or other staff member to come closer to him/her without evoking agitation, the clinician uses the build link technique to strengthen this response. For instance, “after spending time with me in the MSE you seem more relaxed and feel more comfortable with me standing next to you” or “you had a relaxing time today in the MSE, you seem to be in good spirits and more comfortable with your nurse.” Based on learning theory a closer proximal distance in time from MSE exposure to build links outside of the MSE may increase learning potential in the person with dementia [29].

Build link is a technique to promote the transfer of learning from the MSE to the external environment beyond the MSE. The clinician notes the effects of CRB2s via socially rewarding new behavior in the MSE and with other stimuli (environmental and/or interpersonal) outside of the MSE.

Due to gradual learning abilities secondary to dementia, repetition is a central tenant of strengthening the rewarding effects of the MSE and in turn shaping the reward of the MSE to transfer to the clinician, who can then serve as a discriminative stimulus to increase CRB2s in the MSE and outside of the MSE.

(2) $$\begin{array}{cc} & \text{Confirmation}\;\text{Interaction}\;\to \;\text{Link}\;\to \;\text{Link}\;\text{build}\;\to \;\text{Build}\;\text{Link}\;\to \;\text{Repetition}\\ & \text{Social}\;\text{Reinforcement}\overset{\;\;\;\;\;\;\;\;\;\;\;\;\;\;\;\;\;\;\;\;\;}{\to }\;\;\text{Clinician}\;\text{as}\;\\ & \text{discriminative}\;\text{stimulus}\;\text{for}\;\text{shaping}\;\text{CRB}2\text{s}. \end{array}$$

### 3.10. Interpersonal Interactive Module

The interpersonal interactive module is introduced after an elder has experienced the earlier phases of treatment. This module of treatment focuses on building and boarding [30] the interpersonal experience between the caregiver and the elder (social reinforcement), utilizing the contrived reward of the MSE to evoke natural social contingencies. Humanistic approaches are utilized by conceptualizing the MSE as a setting event to evoke and develop reciprocal interactions with the person with dementia based upon his/her functional level (verbal) (nonverbal). Integrated emotion-oriented care (IEC) [27] is a Dutch model which incorporates verbal and nonverbal constructs of nurturance, affirmation, validation of the person's experiences joining in his/her experience (feelings, thoughts, behaviors), and evoking aspects of their personhood.

The following is an example of integrating FAMSET and IEC techniques with a person diagnosed with dementia and the behavioral disturbance of agitation and aggression toward others; especially when people such as staff at a skilled nursing facility would come close to provide care.

Clinician: “you seem to enjoy the shimmering colors and holding the colored light spray (confirmation interaction, validation).”

Elder: “oh, yes”

Clinician: “I notice that you are making a sewing stitch out of this, it looks grand with the change of colors (social reinforcement).”

Elder: “I used to make a lot of sweaters and socks.”

Clinician “Neat, who did you make them for (affirmation, personhood work)?”

Elder: “I made them for my husband and for kids.”

Clinician “What types of colors did you use, any like we have here clinician points at colored light spray (directed interaction)?”

Elder: “I like fun colors.”

Clinician: “I notice that as you stitch and talk to me more about knitting you seem more relaxed (CRB2).”

Elder: “I like this, see this is a box stitch.”

Clinician: “So you would use a box stitch to make sweaters and socks for your husband and kids, seems like a very creative and meaningful activity that benefited your family. How else did you assist your family?” (Affirmation, validation and personhood work).

This interaction script is designed to demonstrate how a clinician identifies intervention choice points within an MSE session and selects the techniques that match the nature of the interaction and strengthen further verbal responding and shaping of behavioral improvements in the person receiving treatment.

The interpersonal interactive module occurs in the latter phase of treatment after prior modules have been experienced by the person with dementia. The MSE is a reward platform for evoking the personhood of the person with dementia and for the clinician to utilize humanistic approaches that nurture, affirm, and validate the elder to increase the experience of connectedness between the clinician and the person with dementia.

## 4. Conclusion

The rationale for this paper is to address the missing gap between outcome research and the clinical process that occurs during an MSE session. The FAMSET framework has been developed to addresses assessment and in-session techniques which include the integration of behavioral and humanistic interpersonal approaches to MSE work. The therapy uses the contrived reward of the MSE as an initial means of evoking well-being in the person with dementia to facilitate interpersonal connectedness between the elder and the clinician. FAMSET uses module work with the person with dementia to foster a positive experience in the MSE. The modules are designed to create boundaries within the treatment thus delineating the therapy into different stages (assessment, following and directing, exploration, random interaction, color form aesthetic response, calm and secure, directed interaction and interpersonal interactive). Using functional analysis, the behavioral disturbances displayed by the person with dementia (CRBs) are the primary focus of the intervention. The goal of the treatment is to evoke CRB2s in the person with dementia by utilizing the positive effects of noncontingent sensory reward combined with the use of shaping and desensitization techniques to strengthen CRB2s in session and promote the transfer of new behavior to interpersonal interactions and, in turn, the external environment.
